# [^18^F]FMeNER-D2: Reliable fully-automated synthesis for visualization of the norepinephrine transporter

**DOI:** 10.1016/j.nucmedbio.2013.08.007

**Published:** 2013-11

**Authors:** Christina Rami-Mark, Ming-Rong Zhang, Markus Mitterhauser, Rupert Lanzenberger, Marcus Hacker, Wolfgang Wadsak

**Affiliations:** a Radiochemistry and Biomarker Development Unit, Division of Nuclear Medicine, Department of Biomedical Imaging and Image-guided Therapy, Medical University of Vienna, Austria; b Department of Inorganic Chemistry, University of Vienna, Austria; c Molecular Imaging Center, National Institute of Radiological Sciences, Chiba, Japan; d Hospital Pharmacy of the General Hospital of Vienna, Austria; e Department of Psychiatry and Psychotherapy, Medical University of Vienna, Austria

**Keywords:** Norepinephrine transporter, PET, Radiosynthesis, Fluorine-18, FMeNER, FMeNER-D2, Automation

## Abstract

**Purpose:**

In neurodegenerative diseases and neuropsychiatric disorders dysregulation of the norepinephrine transporter (NET) has been reported. For visualization of NET availability and occupancy in the human brain PET imaging can be used. Therefore, selective NET-PET tracers with high affinity are required. Amongst these, [^18^F]FMeNER-D2 is showing the best results so far. Furthermore, a reliable fully automated radiosynthesis is a prerequisite for successful application of PET-tracers.

The aim of this work was the automation of [^18^F]FMeNER-D2 radiolabelling for subsequent clinical use. The presented study comprises 25 automated large-scale syntheses, which were directly applied to healthy volunteers and adult patients suffering from attention deficit hyperactivity disorder (ADHD). Procedures: Synthesis of [^18^F]FMeNER-D2 was automated within a Nuclear Interface Module. Starting from 20–30 GBq [^18^F]fluoride, azeotropic drying, reaction with Br_2_CD_2_, distillation of 1-bromo-2-[^18^F]fluoromethane-D2 ([^18^F]BFM) and reaction of the pure [^18^F]BFM with unprotected precursor NER were optimized and completely automated. HPLC purification and SPE procedure were completed, formulation and sterile filtration were achieved on-line and full quality control was performed.

**Results:**

Purified product was obtained in a fully automated synthesis in clinical scale allowing maximum radiation safety and routine production under GMP-like manner. So far, more than 25 fully automated syntheses were successfully performed, yielding 1.0–2.5 GBq of formulated [^18^F]FMeNER-D2 with specific activities between 430 and 1707 GBq/μmol within 95 min total preparation time.

**Conclusions:**

A first fully automated [^18^F]FMeNER-D2 synthesis was established, allowing routine production of this NET-PET tracer under maximum radiation safety and standardization.

## 1 Introduction

The norepinephrine transporter (NET) is one of the major targets in neuropsychiatric and neurodegenerative diseases like attention deficit hyperactivity disorder (ADHD), depression, Alzheimer’s disease (AD), Parkinson’s disease (PD) and substance abuse [1]. For treatment of these diseases, selective norepinephrine (NE) reuptake inhibitors (SNRI) are commonly used, which are typically based on reboxetine. The NET itself facilitates the synaptic reuptake of NE from the synaptic cleft at the presynaptic terminals. Blocking of this transporter prolongs the NE action in the synapse, due to an increase in the concentration of NE in the cleft. Perturbation of the noradrenergic system (and the NET-expression) has been reported to trigger many neuropsychiatric disorders and neurodegenerative diseases. Especially, in the locus coeruleus (LC) a reduction of NET levels has been shown in major depression, AD and PD [1], [2], [3]. Furthermore, also in ADHD a dysregulation of the NE system was reported [2].

For gaining insight in NET availability and dynamics in both healthy and diseased human brains, a non-invasive molecular imaging protocol has been developed using positron emission tomography (PET). Thus, specific and selective NET-PET radioligands are needed. One of the major prerequisites on these candidate tracers is their affinity towards NET, especially when considering the very low density of NET in cerebellum, striatum and human insular cortex [3], [4], [5], [6], [7], [8]. Visualization of NET-rich regions like LC, where NET density is 4–8-fold higher, can be also achieved using ligands with slightly lower affinity [4], [7], [9]. Besides, affinity of the candidate ligands correlates with their binding kinetics, i.e. longer equilibration times for high-affinity substances (e.g. [^125^I]iodo-nisoxetine (Ki = 0.7 nM) reaches its binding equilibrium 3 h post injection) [10], [11]. Moreover, selectivity of the tracers towards NET is required for feasible NET-PET imaging, since NET displays a high similarity to dopamine transporter (DAT) and serotonin transporter (SERT), but shows a significantly lower density in the human brain [5], [12], [13].

**Fig. 1:**
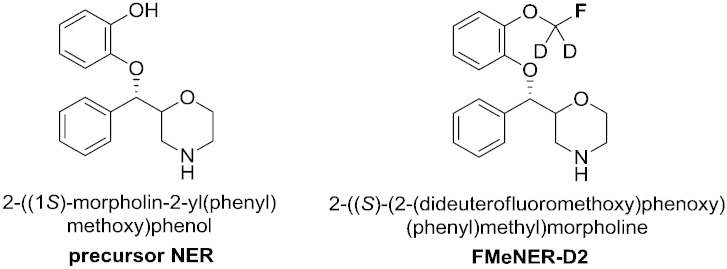
Structures of NER and FMeNER-D2.

Most described NET-PET tracers used in clinical studies are derived from reboxetine as known NET-selective compound. Both ^11^C-methylated and ^18^F-fluoroethylated tracers were proposed [9], [14], [15], [16], [17], [18], [19], [20]. Thereof, [^18^F]FMeNER-D2 ((*S,S*)-2-(α-(2-[^18^F]fluoro[^2^H_2_]methoxyphenoxy) benzyl)morpholine) is so far displaying best properties regarding affinity, selectivity and stability. [^18^F]FMeNER-D2 was developed at the Karolinska Institute (Sweden) in 2004 by Schou and co-workers (Fig. 1) [21], [22], [23].

Major drawbacks of this NET-PET tracer were the complexity of the radiosynthesis and lack of an automated preparation sequence. Thus the aim of this work was the set-up of a reliable automated production of [^18^F]FMeNER-D2 allowing for human NET-PET studies. With this computer-controlled fully-automated synthesis, more than 25 patients were injected with the so-produced [^18^F]FMeNER-D2 at a dose of 4.7 MBq/kg body weight and, so far, subsequent NET PET scans were successfully acquired.

## 2 Materials and methods

### 2.1 Materials

Precursor (*S,S*)-NER (=(*S,S*)-norethyl-reboxetine, =(2*S,* 3*S*)-2-[α-(2-hydroxyphenoxy) benzyl]morpholine) and reference standard (*S,S*)-FMeNER-D2*TFA ((*S*,*S*)-2-[α-(2-(dideutero fluoromethoxy)phenoxy)benzyl]morpholine trifluoroacetate) were obtained from PharmaSynth AS (Tartu, Estonia).

Acetonitrile (ACN for synthesis of DNA, ≥ 99.9% (GC) and ACN HPLC grade), dimethylformamide (DMF, p.a, dried over molecular sieves (4 Å)), dibromomethane-d2 (99 atom% D, copper stabilized), sodium hydroxide, methanol (MeOH, CHROMASOLV®, for HPLC, ≥ 99.9%), ammonium formate, Kryptofix K2.2.2 (4,7,13,16,21,24-hexaoxa-1,10-diazabicyclo[8.8.8]hexacosane) and ethanol (absolute) were purchased from Sigma-Aldrich (Vienna, Austria). Anion-exchange cartridges (PS-HCO_3_) for [^18^F]fluoride fixation were purchased from Macherey-Nagel (Dueren, Germany). Sterile water was purchased from Meditrade Medicare Medizinprodukte (Kufstein, Austria). Phosphate buffer (125 mM) was prepared by dissolving 0.224 g sodium dihydrogen phosphate-monohydrate and 1.935 g disodium hydrogen phosphate-dihydrate (both from Merck, Darmstadt, Germany) in 100 mL sterile water. For solid phase extraction C18 plus SepPak® cartridges and Silica plus long SepPak® cartridges were purchased from Waters (Waters® Associates Milford, USA). For formulation of the product 0.9% saline solution from B. Braun (Melsungen, Germany), 3% saline solution (Landesapotheke Salzburg, Austria) and 125 mM Phosphate buffer) were used. Low-protein binding Millex® GS 0.22 μm sterile filters were obtained from Millipore (Bedford, USA). All other chemicals and solvents for the syntheses and radiosyntheses were obtained from Merck (Darmstadt, Germany) and Sigma-Aldrich with at least analytical grade and used without further purification.

### 2.2 Instrumentation

[^18^F]Fluoride was produced within a GE PET trace cyclotron via ^18^O(p,n)^18^F reaction (16.5-MeV protons; GE Medical Systems, Uppsala, Sweden). H_2_^18^O (HYOX18; > 98%) was obtained from Rotem Europe (Leipzig, Germany).

Evaluation of reaction conditions was performed manually in a lead-shielded hood with small quantities of initial radioactivity (< 1 GBq). After optimization, [^18^F]FMeNER-D2 synthesis was automated within a Nuclear Interface synthesizer (GE Healthcare, Sweden), remotely controlled by a standard laptop with dedicated processing software (Fig. 3).

Purification of [^18^F]FMeNER was performed by semi-preparative reversed phase HPLC using the built-in semi-preparative HPLC system equipped with a radioactivity-, a UV-detector (Linear Instruments Model 200 Detector UV/VIS) and a LaPrep HPLC pump (VWR International, Radnor, USA). A Phenomenex® Gemini, C-18 column with TMS endcapping, 10 μm, 250 × 10 mm (Phenomenex®, Aschaffenburg, Germany) and a mobile phase of MeOH/0.1 M ammonium formate (AMF) in water 50/50 v/v% at a flow rate of 12 mL/min was used for purification.

Analytical HPLC was performed on Merck-Hitachi LaChrom HPLC system (L-7100 pump; LaChrom L-7400 UV detector at 254 nm) and a NaI radio-detector (Bertholdt Technologies, Bad Wildbach, Germany) using Raytest software (Raytest, Straubenhardt, Germany). A Phenomenex Prodigy Phenyl-PH3 column; 250 × 4.6 mm, 5 μm (Phenomenex®, Aschaffenburg, Germany) and a mobile phase consisting of ACN/0.1 M AMF in water 50/50 %v/v at a flow rate of 2 mL/min was used. The osmolality was measured with a Wescor osmometer Vapro® 5600 (Sanova Medical Systems, Vienna, Austria) and pH was measured using a WTW inoLab 740 pH meter (WTW, Weilheim, Germany). GC-Analysis was performed with a Bruker Gas Chromatography System 430-GC. Radio-TLC Analysis was performed using silica gel 60 RP-18 F_254_S plates from Merck (Darmstadt, Germany) with a mobile phase consisting of ACN/water 70%/30% v/v. Analyses of radio-TLC plates were done using a Canberra-Packard Instant Imager (Perkin Elmer, Watford, UK).

PET scans were performed in the Department of Nuclear Medicine, Medical University Vienna, using a GE Advance PET-scanner and kinetic model was done with PMOD 3.0 using a two compartment model.

## 3 Methods

### 3.1 Preparation of 1-bromo-2-[^18^F]fluoromethane-d2 ([^18^F]BFM)

**Fig. 2:**
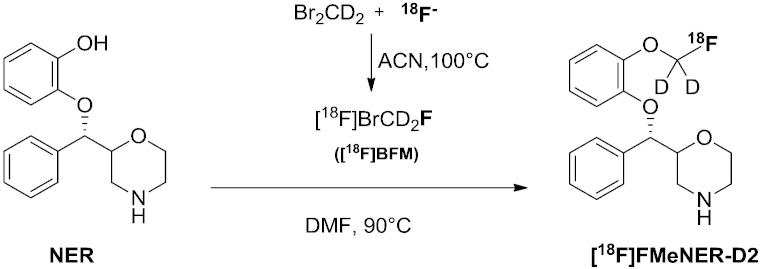
Reaction scheme for [^18^F]FMeNER-D2.

In Fig. 2 the reaction scheme for the synthesis of [^18^F]BFM and [^18^F]FMeNER-D2 is outlined.

[^18^F]Fluoride was produced within the cyclotron via ^18^O(p,n)^18^F reaction and trapped on an anion exchange cartridge (PS-HCO_3_). It was eluted from the cartridge using 0.8 mL of solution containing (20 mg/mL, 53.2 μmol/mL) Kryptofix 2.2.2 and (4.5 mg/mL, 33.2 μmol/mL) K_2_CO_3_ in ACN/water 80%/20% v/v. To test the influence of the quality of the water used on the specific activity ‘trace select water’ was compared with compared with ‘aqua ad injectabilia’.

After complete evaporation, azeotropic drying was performed iteratively by twofold addition of 0.8 mL ACN. For optimization of [^18^F]BFM synthesis, the dried fluoride was split and cooled to 20 °C and 50 μL dibromomethane-d2 in 500 μL ACN, DMF or *o*-dichlorobenzene was added. The vessel was sealed tightly and the mixture heated (90 °C, 100 °C) for 5 min. The crude solution containing [^18^F]fluoride, dibromomethane-d2 and [^18^F]BFM was cooled to RT and distilled under different conditions for optimization purpose: a smooth He stream (10 ml/min, 30 mL/min and 40 mL/min) was applied over (*a*) an empty, sealed 2 mL glass vial; (*b*) 2 Omnifit cartridges filled with silica in sequence; and (*c*) 4 silica plus SepPak® cartridges linked in series. The volatile [^18^F]BFM was trapped in a second vessel containing 400 μL DMF (at RT or cooled at − 20 °C in advance). The chemical and radiochemical purity of the product was assessed by analytical HPLC. The [^18^F]BFM solution was directly used for the next step of synthesis.

### 3.2 Synthesis of [^18^F]FMeNER-D2

Synthesis was performed according to Schou et al [22]. Precursor NER (1 mg; 3.5 nmol) was dissolved in 200 μL DMF and 6 μL of aqueous 5 M NaOH was added. After addition of 400 μL of the freshly produced [^18^F]BFM in DMF, the reaction vessel was sealed and the mixture heated to 90 °C for 5 min. The crude reaction mixture was analyzed by analytical HPLC and TLC.

### 3.3 Automation of radiosynthesis

Automation of synthesis was done within a Nuclear Interface module using the optimized conditions.

**Fig. 3:**
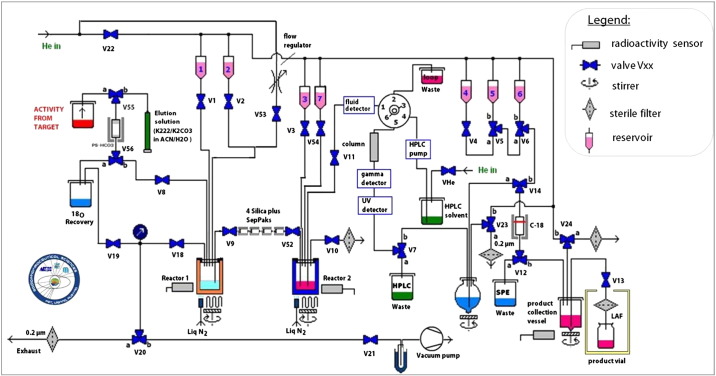
Set-up of the automated process of [^18^F]FMeNER-D2 synthesis.

[^18^F]Fluoride (20–30 GBq) was transferred into a lead shielded hot cell and connected to the V55 target water line (Fig. 3). [^18^F]F^−^ was sucked over the anion exchange cartridge using a vacuum pump (V55 →a, V56 →a, V19, V20, V21→b, vacuum pump) and the ^18^O enriched water recovered. [^18^F]Fluoride was eluted from the PS-HCO3 cartridge into the reactor 1 by priming (via V55→b, V56→b, V8, V18, V20→b, V21 and vacuum pump) of 0.8 mL elution solution containing K 2.2.2 and potassium carbonate in ACN/trace select water 80/20). Then, [^18^F]fluoride was azeotropically dried by heating to 100 °C for 5 min. Drying was completed by elevation of temperature to 120 °C for 2 min. Then, the vessel was cooled to 40 °C and 0.8 mL ACN was added over the same lines as the elution solution. After drying, another portion of ACN was added from vial 1 (using Helium from V22) and evaporated to complete dryness. The thoroughly dried fluoride was cooled to 20 °C and dibromomethane-d2 (50 μL) in 500 μL ACN was added via vial 2. The vessel was sealed tightly and the mixture heated to 100 °C for 5 min. After cooling reactor 1 to 28 °C, the second reactor containing 400 μL DMF was cooled to − 20 °C. Purification by distillation was performed over 4 silica plus SepPak® cartridges using a smooth He stream (1 min with 10 mL/min; then 40 mL/min for 10–15 min) and the pure [^18^F]BFM trapped in reactor 2 (by bubbling inside the DMF through a peek tubing (**V52**)). After a plateau of activity in the second reactor was reached, distillation was stopped and precursor NER (1 mg in 200 μL DMF + 6 μL 5 M NaOH) was transferred into **reactor 2** via **vial 3**. The reaction mixture was sealed and alkylation was performed for 5 min at 90 °C. The reaction was cooled to RT and quenched by addition of 1 mL water (**vial 7**). The crude product solution was transferred to the prep HPLC injection loop and automatically injected onto the column (Phenomenex Gemini, MeOH/0.1 M AMF 50%/50% v/v; 12 mL flow rate) using a fluid detector. The product fraction was cut into a 100 mL bulb and diluted with 80 mL water. After removal of solvents by reversed-phase SPE (C-18 plus SepPak) [24], the product was washed with 10 mL water (**vial 6**) and eluted with 1.5 mL EtOH (**vial 5**) into the product collection vessel containing 4 mL 0.9% saline, 1 mL 125 mM phosphate buffer (pH 7.4) and 1 mL 3% saline. The tubings were washed with another 5 mL saline (**vial 4**) and the formulated product was passed over a 0.22 μm sterile filter into an evacuated sterile vial containing a further 5 mL 0.9% saline. Final volume was 17.5 mL, containing 8.5% ethanol. Purified [^18^F]FMeNER-D2 was analyzed by analytical HPLC, GC and TLC; pH and osmolality were measured and a y-spectrum was recorded. Testing for endotoxins and sterility was performed retrospectively. Total quality control was performed according to the guidelines presented in the European Pharmacopoeia and using routine procedures at the PET Centre of the Vienna General Hospital, Medical University of Vienna. Specific radioactivity was assessed by quantification of the non-radioactive product (HPLC UV channel at 254 nm) and determination of overall radiochemical yield (GBq at end of synthesis).

## 4 Results

### 4.1 Preparation of 1-bromo-2-[^18^F]fluoromethane-d2 ([^18^F]BFM)

Small scale reactions evinced that reaction of [^18^F]fluoride with dibromomethane-d2 was highly dependent on the solvent used. Using DMF as solvent did only result in 5%–25% [^18^F]BFM, even at elevated temperatures (90–150 °C) only 75%–95% of [^18^F]fluoride was recovered. Nucleophilic substitution in o-DCB at 100 °C, commonly used for synthesis of 1-bromo2-[^18^F]fluoroethane [25], [26], [27], [28], evinced 52% ± 5% radiochemical incorporation. Interestingly, a second radioactive byproduct was formed, eluting from the HPLC at almost the same retention time as the later formed [^18^F]FMeNER-D2. Synthesis of [^18^F]BFM in ACN for 5 min at 100 °C showed the best results; 56% ± 4% radiochemical incorporation was found without by-product formation.

Purification of the crude [^18^F]BFM is mandatory, and was performed by distillation. For the removal of ACN and dibromomethane-d2 three different approaches were tested. At first, an empty 2 mL glass vial was linked in between the crude (**reactor 1**) and the DMF trap (**reactor 2**). Unfortunately, almost all of the product was retained within this trap, and only very small amounts of [^18^F]BFM could be obtained in the DMF distillate. Also elevation of temperature from RT to 50 °C did not result in much higher activity in the distillate; on the contrary, larger quantities of ACN were spilled into the product trap. Since it was crucial to remove ACN for the next reaction step, this set-up was discarded. Next, two Omnifit cartridges (3 mm/25 mm each) were filled with silica (80–100 mesh, each 300 mg) and secured with glass wool and frits. These were linked in series and inserted between the crude product vial and the DMF trap. Hereby, all ACN and dibromomethane were trapped on the columns, but also almost the entire product was retained on the column. The quantities of product within the DMF trap were too small for further reactions. Therefore, a third approach of purification was tried: linking 4 silica plus SepPak® cartridges in series. Using this setup, the removal of ACN and dibromomethane-d2 could be achieved at RT, and also the amounts of trapped [^18^F]BFM (> 97% radiochemical purity) were sufficient for further syntheses. After 10–15 min, no further product was distilled using 40 mL/min He.

### 4.2 Synthesis of FMeNER-D2

**Fig. 4:**
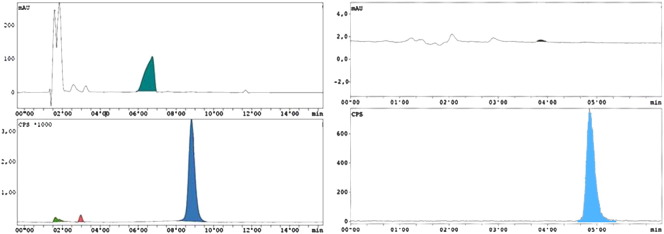
Preparative and analytical HPLC chromatogram of [^18^F]FMeNER-D2.

The distilled [^18^F]BFM (in 400 μL DMF) was reacted with 1 mg NER in 200 μL DMF and 6 μL 5 M NaOH yielding 95%–99% radiochemical incorporation after 5 min at 90 °C. For purification, a preparative HPLC assay was developed and allowed the separation of all compounds. The retention times were 1.62 min (k’ = 0) for [^18^F]fluoride, 2.50 min (k’ = 0.55) for precursor NER, 2.97 min (k’ = 0.84) for [^18^F]BFM, 6.77 min (k’ = 3.19) for Br_2_CD_2_ and 8.83 min (k’ = 4.46) for [^18^F]FMeNER-D2. Less than 5% of radioactive impurities were detected in all [^18^F]FMeNER-D2 syntheses, so far. After dilution of the product fraction (8–9.5 min) with 80 mL water, SPE was performed using a C18 plus SepPak®. The product was formulated (8.5% EtOH), sterile filtered and analyzed by analytical HPLC. The retention times were 1.49 min (k’ = 0) for [^18^F]fluoride, 3.03 min (k’ = 0.66) for [^18^F]BFM, 3.62 min (k’ = 0.99) for Br_2_CD_2_, 4.03 min (k’ = 1.21) for precursor NER and 5.42 min (k’ = 1.98) for product [^18^F]FMeNER-D2, respectively. In Fig. 4, exemplary HPLC chromatograms (both preparative and analytical) are shown.

### 4.3 Automation of synthesis

Full automation of [^18^F]FMeNER-D2 synthesis was achieved successfully. [^18^F]Fluoride fixation, azeotropic drying and reaction with Br_2_CD_2_ were performed with high yields and purity. Automation of distillation of [^18^F]BFM remained the crucial step within the synthesis, where the major loss of activity occurred. About half of the formed [^18^F]BFM was found to be retained on the Silica plus cartridges when distilling with 40 mL/min He stream. However, the amount of [^18^F]BFM trapped using 40 mL/min was sufficient for the following reaction and purification. Increasing the flow to 50 mL/min and higher, did on the one hand result in higher activity trapped in the second reactor, but separation from dibromomethane-d2 and ACN could no longer be achieved quantitatively. Subsequently, almost quantitative conversion of [^18^F]BFM to FMeNER-D2 was observed for the automated large scale preparations.

So far, more than 25 fully automated radiosyntheses have been performed, yielding 1.53 ± 0.6 GBq [^18^F]FMeNER-D2 (12.0% ± 5.2%, not corrected for decay) within 100 min from the end of bombardment (for details see Table 1). Specific radioactivities ranged from 430 to 1707 GBq/μmol. Radiochemical and chemical purity was always ≥ 98%, osmolality and pH were found to be in a physiological range. GC analysis evinced ACN < 10 ppm and methanol < 20 ppm for each batch. In TLC and analytical radio-HPLC presence of [^18^F]fluoride and [^18^F]BFE was quantified, and found to be < 1%.

**Table 1:** Fully automated preparation of [^18^F]FMeNER-D2: Yields, loss of radioactivity and required time.

n ≥ 15	GBq	% of initial activity (corr. for decay)	Δt to start of synthesis [min]
[^18^F]fluoride starting activity in reactor 1	25.2 ± 4.3	100	0
pure [^18^F]BFM after distillation trapped in reactor 2	3.6 ± 1.1	23.6 ± 6.9	59 ± 4
residual after HPLC injection in reactor and loop waste	1.2 ± 0.3	7.7 ± 1.9	76 ± 1
[^18^F]FMeNER-D2 final product yield	1.53 ± 0.6	12.0 ± 5.2	95 ± 6
Specific activity [GBq/μmol]	598.8 ± 352 (range 432.7–1707.4)		

**Fig. 5:**
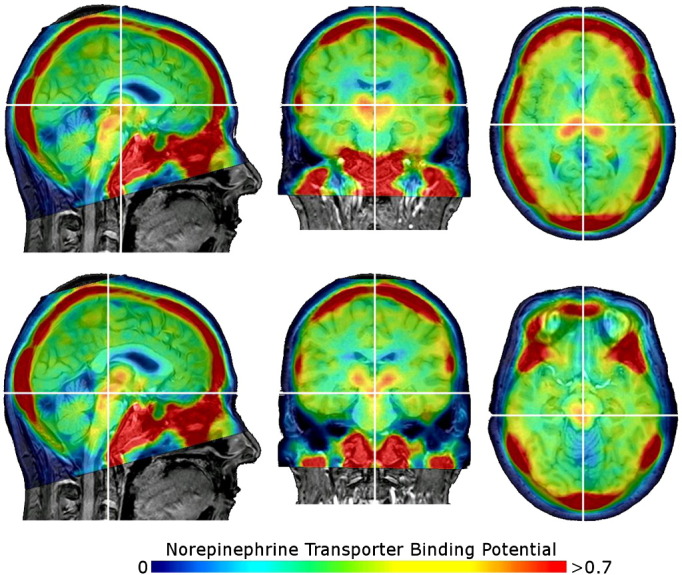
Parametric voxel-wise binding potential (BP_ND_) maps of the norepinephrine transporter superimposed on structural magnetic resonance images in a healthy subject (male, age 21 years) using [^18^F]FMeNER-D_2_. The white cross in the triplanar view indicates the thalamus (upper column) and the locus coeruleus (lower column), the colour bar indicates the norepinephrine transporter binding potential_._ The BP_ND_ has been calculated using a 2-tissue compartment model with the caudate as reference region and the software PMOD 3.0. The injected dose was 416.6 MBq, the specific activity was 520.6 GBq/μmol (corrected for application time). Note also the high uptake in extracerebral areas.

For the ongoing clinical study, the product was injected directly after release (4.7 MBq/kg body weight) to healthy volunteers and ADHD patients. 120 min post injection, dynamic PET scans were performed for 60 min. In Fig. 5, exemplary NET distribution maps measured with PET and FMeNER-D2 in a health male subject are shown (for details, see figure’s legend).

## 5 Discussion

Since so far only [^18^F]FMeNER-D2 is used as a feasible NET-PET tracer in clinical trials, an automated radiosynthesis is of great benefit in terms of routine availability, reproducibility and radiation safety.

For evaluation of optimum parameters, small scale experiments were performed. During the examinations, the purification step of [^18^F]BFM turned out to be the chokepoint of the synthesis. Within the first experiments, it rapidly turned out that distillation at 70 °C as described by Schou et al. was not the method of choice. Thus, huge amounts of ACN were found to be present in the DMF trap even when very weak adjuvant gas flow was applied and no matter which trap was used (glass vial, Silca plus SepPak® and omnifit cartridges). The presence of ACN, even in trace amounts, seems to impede the reaction between NER and [^18^F]BFM, possibly due to the insolubility of NER in ACN. Moreover, large quantities of Br_2_CD_2_ were found in the DMF trap as well, leading to cold side product formation (BrMeNER) with unknown, but expected high affinity towards NET. Therefore, a milder distillation procedure needed to be established, and was met when applying 28 °C. This temperature seems to be high enough for the highly volatile synthon [^18^F]BFM to be drawn by distillation, but low enough to prevent evaporation of Br_2_CD_2_ and ACN. Thereby an auxiliary gas stream of 40 mL/min was found to be optimal. Best results were obtained when using 4 Silica plus SepPak® cartridges linked in series.

Upon complete separation of ACN from the pure [^18^F]BFM solution, reaction with precursor NER yielded > 95% radiochemical incorporation of [^18^F]FMeNER-D2 in crude mixture. The purification of the product from [^18^F]BFM and NER was performed quantitatively using the proposed preparative HPLC assay with a retention time of 8–9 min for [^18^F]FMeNER-D2. After SPE (using a C18 plus SepPak), pure [^18^F]FMeNER-D2 was eluted almost quantitatively from the cartridge, hence only 0.5%–2% of the pure product was bound irreversibly. Satisfying amounts of product were achieved after formulation and sterile filtration. Strict radiopharmaceutical quality control was passed for all synthesized batches allowing for preclinical testing and *in*-*vivo* applications. Analytical HPLC evinced high specific activities (SA) for all lots of [^18^F]FMeNER-D2 produced. High variation (430–1707 GBq/μmol) in SA was observed but can be explained likewise: The amount of non-radioactive FMeNER-D2 present in the product was so small that it was below the limit of detection. Therefore, the SA was only dependent on the product yield. Hence, the given values represented the lower limits in achieved SA, leading to the observed variances.

Furthermore, we examined the influence of the quality of water used for the fluoride elution solution in preliminary tests. We compared elution solution with ‘aqua ad injectabilia’, commonly used for other tracer preparations, with the one made with ‘trace select water’. Specific activity was found to be much lower (139.9 ± 69 GBq/μmol; range: 57.8–231 GBq/μmol, n ≥ 8) for ‘aqua ad inj.’ as compared to the experiments with elution solutions containing ‘trace select water’ (SA = 598.9 ± 352 GBq/μmol; range: 432.7–1707 GBq/μmol n ≥ 15).

## 6 Conclusion

Automation of [^18^F]FMeNER-D2 synthesis was set-up successfully, enabling a reliable and routine preparation of this NET-PET tracer for clinical use. More than 25 fully automated syntheses were performed yielding 1.53 ± 0.6 GBq [^18^F]FMeNER-D2 with high purity and excellent specific radioactivities ready to use for animal or human application. Enhancing the routine availability of [^18^F]FMeNER-D2 will lead to an increased interest in clinical trials regarding the norepinephrine transporter using PET.
